# Supplementary material for: Splice-Junction-Based Mapping of Alternative Isoforms in the Human Proteome
Source: Cell Rep. Author manuscript; Available in PMC 2020 Jan 15. (PMC6961840; doi:10.1016/j.celrep.2019.11.026)
Supplement: 2 [file NIHMS1546469-supplement-2.zip › DF1/PXD009737/TestisTrypsin/TestisTrypsin_46_RASSF7_EVAAAPAPEWCPLAAQPQASGSGAGGAEPR.pdf]

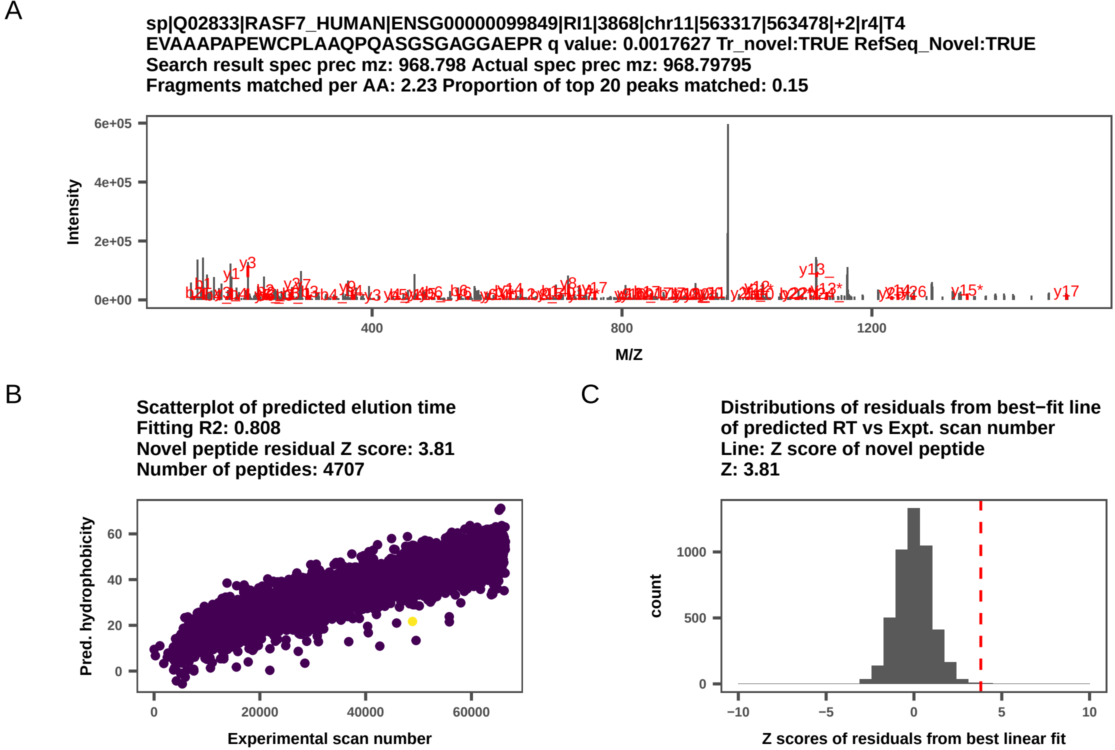
A
sp|Q02833|RASF7_HUMAN|ENSG00000099849|RI1|3868|chr11|563317|563478|+2|r4|T4
EVAAAPAPEWCPLAAQPQASGSGAGGAEPR q value: 0.0017627 Tr_novel:TRUE RefSeq_Novel:TRUE
Search result spec prec mz: 968.798 Actual spec prec mz: 968.79795
Fragments matched per AA: 2.23 Proportion of top 20 peaks matched: 0.15
Intensity
6e+05
4e+05
2e+05
0e+00
M/Z
400
800
1200
y3
y1
y13_
y15*
y17
B
Scatterplot of predicted elution time
Fitting R2: 0.808
Novel peptide residual Z score: 3.81
Number of peptides: 4707
Pred. hydrophobicity
60
40
20
0
Experimental scan number
0
20000
40000
60000
C
Distributions of residuals from best−fit line
of predicted RT vs Expt. scan number
Line: Z score of novel peptide
Z: 3.81
count
1000
500
0
Z scores of residuals from best linear fit
−10
−5
0
5
10
